# Adaptation to Changes in Higher-Order Stimulus Statistics in the Salamander Retina

**DOI:** 10.1371/journal.pone.0085841

**Published:** 2014-01-21

**Authors:** Gašper Tkačik, Anandamohan Ghosh, Elad Schneidman, Ronen Segev

**Affiliations:** 1 Institute of Science and Technology Austria, Klosterneuburg, Austria; 2 Indian Institute of Science Education and Research-Kolkata, Mohanpur (Nadia), India; 3 Department of Neurobiology, Weizmann Institute of Science, Rehovot, Israel; 4 Faculty of Natural Sciences, Department of Life Sciences and Zlotowski Center for Neuroscience, Ben Gurion University of the Negev, Be'er Sheva, Israel; Bascom Palmer Eye Institute, University of Miami School of Medicine; United States of America

## Abstract

Adaptation in the retina is thought to optimize the encoding of natural light signals into sequences of spikes sent to the brain. While adaptive changes in retinal processing to the variations of the mean luminance level and second-order stimulus statistics have been documented before, no such measurements have been performed when higher-order moments of the light distribution change. We therefore measured the ganglion cell responses in the tiger salamander retina to controlled changes in the second (contrast), third (skew) and fourth (kurtosis) moments of the light intensity distribution of spatially uniform temporally independent stimuli. The skew and kurtosis of the stimuli were chosen to cover the range observed in natural scenes. We quantified adaptation in ganglion cells by studying linear-nonlinear models that capture well the retinal encoding properties across all stimuli. We found that the encoding properties of retinal ganglion cells change only marginally when higher-order statistics change, compared to the changes observed in response to the variation in contrast. By analyzing optimal coding in LN-type models, we showed that neurons can maintain a high information rate without large dynamic adaptation to changes in skew or kurtosis. This is because, for uncorrelated stimuli, spatio-temporal summation within the receptive field averages away non-gaussian aspects of the light intensity distribution.

## Introduction

Adaptation is ubiquitous in the nervous system, from synaptic depression [1], [2] and single neuron spiking [3], [4], to the activity of neural modules (e.g. [5]). In sensory systems, it has been suggested to be a key design principle of the neural code [6], which may allow for optimal information coding by matching the neural responses to stimulus statistics [7], [8], [9], [10]. The retina is one of the most studied highly adaptive neural circuits, in which the mapping between stimuli and neural response changes to match the statistics of the mean light intensity [11], temporal and spatial contrast and spatial scale [12], [13], [14], pattern [15], relative motion [16] and periodicity [17].

Since adaptation requires some form of memory and inference of the stimulus statistics to which the system should adapt, the mechanism and nature of adaptation have been studied extensively. For example, the dynamic structure of the retinal ganglion cell receptive fields [18], and contrast adaptation in the vertebrate and fly visual systems [13], [14], [19], [20], [21], [22] have been characterized as gain-control mechanisms that serve to efficiently encode the variation of the stimulus around the mean into a limited dynamic range of firing rates at the output. It has been further shown that neural systems adapt not only to various stationary stimuli, but also to dynamic changes in stimulus distributions taking place across multiple timescales [23], [24], [25].

Despite its ubiquitous presence, it is still not clear what are the limits to adaptation, and in particular, which stimulus changes should lead to adaptive responses and which should not. This is because adaptation, by its very nature, comes with an inherent caveat or cost: stimuli can no longer be read out from instantaneous responses of an adapting system, but can also involve responses potentially stretching far into the past [25]. Since most studies of adaptation analyzed neural systems' response to first- and second-order spatio-temporal statistics in the stimulus, we addressed here the nature of neural response to changes in higher-order structure of visual stimuli; such higher-order structure is characteristic of natural scenes [26], [27] and is perceptually salient in humans [28], [29], [30].

Spatial textures were used previously to study the responses of cat LGN neurons to stimuli containing higher-order statistical structure [31]. The authors reported that contrast-gain control responds to spatial root-mean-square contrast but not to the higher moments in the pixel luminance distribution. These results raised a number of important questions that we address here: **(i)** are there any signatures of adaptation to higher-order statistics, especially if *spatially uniform* stimuli that match the naturalistic range of skew/kurtosis are used instead of the spatial textures (as used by Ref [31]), which cannot accommodate the same effective range of skewness/kurtosis values; **(ii)** do changes in higher-order stimulus statistics affect the cells' rate of information coding; and finally, **(iii)** what would be theoretically expected changes for LN-type neurons in response to changes in higher-order stimulus statistics if the neurons were maximizing the amount of transmitted information.

To characterize adaptation to stimulus statistics beyond luminance and contrast, we studied retinal responses to spatially uniform stimuli where light intensities were drawn independently from distributions with tunable amounts of skewness and kurtosis. We organized our analysis as follows. First, we report in detail on our choice of stimuli; next, we use 2D linear-nonlinear (LN) models to characterize the cells' responses, and analyze in detail the changes in the linear (L) stage when higher-order statistics change, followed by the analysis of nonlinear (N) stage changes. To assess the functional significance of these changes, we compare them to changes induced during contrast adaptation. We conclude by examining theoretically optimal LN coding of higher-order statistics stimuli, and compare these predictions to data.

## Materials and Methods

### Natural image statistics

To sample the range of naturally occurring values for contrast, skewness and kurtosis, we took a sample of 501 calibrated grayscale images from the Penn Natural Image Database (PNIDb) [32]. From each image we selected 400 random patches 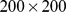 pixels in size, which corresponds in area to the angular size of about 3 degrees, roughly the size of the center receptive field of a salamander retinal ganglion cell. Averaging over each patch to get the mean luminance in that patch, we computed the contrast, skewness and kurtosis of the distribution of patch luminances in a given image. Repeating the process over all images in our selection (containing shots of Baboon habitat in Okavango delta in Botswana, including landscape images, some with horizon, closeups of the ground, and a small selection of man-made objects in that habitat), we accumulated natural distributions of contrast, skewness and kurtosis.

In our analyses, contrast is defined as 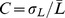, where 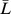 is the mean luminance, 
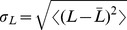
 is the std of the luminance distribution 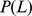 and brackets denote averaging over this distribution; skewness 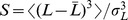; and kurtosis 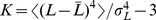. Note that kurtosis is defined to be 0 for Gaussian distributions.

### Electrophysiology

Multi-electrode array recordings were performed on adult tiger salamander (*Ambystoma tigrinum*) [33]. All experiments were approved by the Ben-Gurion University of the Negev Institutional Animal Care and Use Committee and were in accordance with government regulations of the State of Israel. Prior to the experiment the salamander was adapted to bright light for 30 minutes. Retinas were isolated from the eye and peeled from the sclera together with the pigment epithelium. Retinas were placed with the ganglion cell layer facing a multielectrode array with 252 electrodes (Ayanda Biosystems, Switzerland) and superfused with oxygenated (95% O_2_, 5% CO_2_) Ringer medium which contained 110 mM NaCl, 22 mM NaHCO_3_, 2.5 mM KCl, 1 mM CaCl_2_, 1.6 mM MgCl_2_, and 18 mM glucose, at room temperature. The electrode diameter was 10* µ*m and electrode spacing varied between 40 and 80* µ*m. Recordings of 24–30 hours were achieved consistently. Extracellularly recorded signals were amplified (MultiChannel Systems, Germany), digitized at 10 kHz on four personal computers and stored for off-line spike sorting and analysis. Spike sorting was done by extracting from each potential waveform the amplitude and width, followed by manual clustering using an in-house program written in MATLAB (cf. [34]). The quality of spike sorting was monitored by inspection of the inter spike histogram for refractory period violations. In our data sets the majority of the cells, 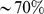, had less than 1% refractory period violations and 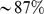 less than 2% violation.

### Stimulation

The stimulus was projected onto the salamander retina from a CRT video monitor (ViewSonic G90fB) at a frame rate of 60 Hz such that each stimulus frame was presented twice in a row (for a stimulus sampling rate of 30 Hz) using standard optics. This rate was chosen because it has been shown previously that it is roughly the slowest rate that is still sufficiently high for linear filter estimation using full field flicker stimulus (i.e., the temporal correlation in the stimulus is not longer than the scale at which the linear filters of salamander retinal ganglion cells change) [35], [36]; consequently this is the most correlated stimulus with a trivial correlation structure (fully correlated in space, maximally slow refresh rate with IID frames in time). The stimulus intensity was presented in grayscale and was gamma corrected for the monitor. All stimulus distributions have the same mean luminance of 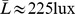. Gaussian stimulus distributions with the desired variance were generated using MATLAB random number generator, with widths of 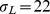 lux for C+ and 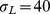 lux for C++. We refer to all non-Gaussian stimuli as HOS (higher-order statistics) stimuli, which we generated with the statistics given in Table 1. All HOS stimuli were constructed as mixtures of two Gaussian components, G1 and G2, whose parameters are specified in Table 2.

**Table 1:** Stimuli 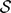 used in the experiment (see main text for the definition of the statistics 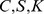).

stimulus 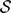	symbol	contrast 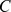	skewness 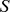	kurtosis 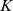
**Gaussian**	C+	**0.097**	0	0
	C++	**0.177**	0	0
**Skewed**	S−−	0.170	−**1.9**	5.1
(HOS)	S−	0.172	−**1.0**	6.0
	S+	0.175	**1.0**	5.2
3	S++	0.178	**1.9**	5.2
**Kurtotic**	K−−	0.177	0	−**1.8**
(HOS)	K−	0.176	0	−**0.9**
	K+	0.173	0	**5.3**

The shorthand symbol for the stimulus starts with the C/S/K (for contrast, skew, kurtosis) and is followed by −,−−,+,++ (small magnitude and negative, large magnitude and negative, small magnitude and positive, large magnitude and positive); therefore, 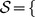
C+,C++,S−−,S−,S+,S++,K−−,K−,K+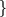. Parameters in the table denoted in bold were varied in each of the three stimulus categories.

**Table 2:** Stimulus generating parameters for HOS stimuli.

stimulus	(std, weight) of G1	(std, weight) of G2	mean G2 - mean G1
S−−	(100, 0.25)	(20, 0.75)	80
S−	(100, 0.25)	(20, 0.75)	32
S+	(20, 0.75)	(100, 0.25)	32
S++	(20,0.75)	(100, 0.25)	80
K−−	(8, 0.5)	(8, 0.5)	80
K−	(8, 0.5)	(8, 0.5)	40
K+	(20, 0.75)	(100, 0.25)	0

HOS stimuli are a mixture of two Gaussian distributions G1 and G2, whose parameters are given in the second and third columns, respectively. The displacement of the mean of the second Gaussian vs the first Gaussian is given in the last column. All units are in lux, and all distributions are matched in mean and have a std of 40 lux. All distributions are 0 outside of the range 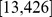 lux, which are the physical limits of the display device.

We performed two experiments. In the first (23 cells), all 9 stimuli were displayed in long, non-repeated sequences (52202 frames of 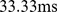 each for each of the 9 stimuli), allowing us to infer LN models precisely; we used the Gaussian stimulus to fit LN models using both the spike-triggered average/covariance and maximally informative dimensions, to check how closely the two inference methods agree. In the second experiment (40 cells), the two Gaussian stimuli were absent, while for the 7 remaining HOS stimuli each non-repeated sequence was followed by a repeated sequence (30 repeats, 602 frames at 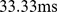 for each repeat), used to validate our models.

### Linear filters

In inferring LN encoding models, reverse correlation techniques cannot directly be applied to non-Gaussian stimuli because they lead to biased filter estimates. Instead, we used maximally informative dimensions (MID) [37]. MID provides unbiased filter estimates that are consistent with the maximum likelihood inference [38]. Moreover, MID extracts the stimulus subspace that is informative about the spike without the need to assume the functional form of the nonlinearity, which is usually required for tractable maximum likelihood estimation of linear filters. Briefly, MID works as follows. To look for a single significant filter 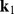, one performs the following maximization over possible linear filters 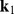, constrained to unit norm (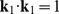): 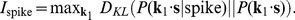
(1)


Here 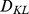 is the Kullback-Leibler divergence [56] between the spike-triggered distribution and the prior distribution of stimulus fragment projections onto 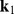, and 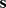 are stimulus fragments (those preceding the spike for the spike-triggered distribution, and all fragments for the prior distribution). To look for 2D models, we repeated the same optimization with two filters 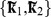; the spike-triggered and prior distributions are two-dimensional in this case. For all neurons, the single most informative filter 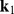 was contained in the space of the 2 filters 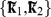; for further analysis, we rotated the system of reference such that the first filter was the single most informative filter 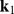 (which mostly corresponded to the spike-triggered average for those cells that were exposed to Gaussian stimulus), while the second filter 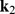 spanned the 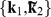 subspace together with 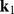, and formed an orthonormal basis, 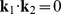, 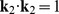.

The filters extended over 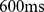 and were sampled on 36 equidistant points, with temporal resolution of 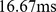. We expressed the filters as a combination of 16 basis functions, 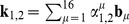, where 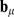 are unit-area Gaussian bumps with 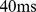 width, uniformly tiling the 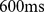 span of the filters, and 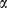 are the expansion coefficients; we maximized 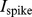 in the space of parameters 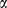. This expansion made the filters smooth and very slightly improved generalization performance, but the results were stable even if we inferred directly in the space of 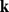.

For performance reasons we estimated 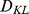 during MID optimization runs using kernel-smoothing estimation; for final results we used the context-weighted-tree (CTW) estimator [39]; the two estimators agreed without bias and to within 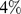 for final filters across all cells and stimuli. Optimization was done using custom stochastic gradient descent code that can avoid local maxima. We performed two optimization runs for each cell and each stimulus, and the values of information per spike between the two runs differed by 1% on average, 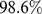 of the runs had a difference smaller than 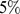.

To quantitatively compare the shapes of the filters across stimuli in experiment 1, we needed to ensure that each stimulus condition had enough spikes for good filter inference. We required each cell to have an average firing rate of at least 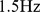; 15 out of 23 cells passed this cut. This threshold, as we explain below, provided us with enough spikes such that an estimation of linear filters using MID is very reliable for a synthetic LN benchmark model in which true filters are known. In experiment 1 we displayed Gaussian stimuli in addition to HOS stimuli, and we computed spike-triggered average/covariance to extract STA and the next most significant filter (orthogonal to the STA) from Gaussian segments. To judge the significance of the eigenvectors in the STC analysis we used bootstrapping with subsets of recorded spikes following [40].

To estimate an error on our determination of linear filters, and specifically on the balance index 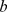, we performed two analyses. First, we made two independent runs of MID for each cell and condition to find the linear filters. The overlap (scalar product) of the normalized filters across all cells and conditions was 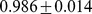 (min 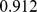, max 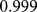) in the two MID runs. Then we compared the spread between the values of 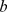 extracted from these filters. On average the runs differ by 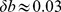. These errors are estimates due to stochastic optimization used to implement MID inference (note that across the two independent runs, the stimulus and spike trains are exactly the same). Second, to ask about the error due to the limited number of spikes, we ran our MID procedure on 20 spike trains independently generated by a synthetic LN model for which the true filter was known; the number of spikes generated corresponded to the number of spikes in our cells that just passed the selection threshold. This error amounted to 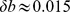 (with an average filter overlap between reconstructed linear filters always 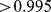), which includes the error due to stochastic optimization for the synthetic model. These considerations suggest that stochastic optimization is likely the dominant source of error for our inference, and that this error is significantly below the variations in the balance index due to the stimulus condition.

### Nonlinearities and PSTH prediction

After having reconstructed the linear filters 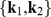, we estimated the nonlinearities as follows: 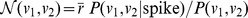, where 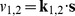 are the projections of the stimulus onto the two linear filters, and 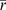 is the mean firing rate of the neuron in a given stimulus condition. For 2D nonlinearities, we binned 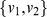 values on a 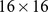 grid; for estimating 1D projections of the full 2D nonlinearity, we binned into a number of bins that was adaptively dependent on the number of spikes, and used kernel smoothing to approximate the probability distributions. Prediction performance was only slightly changed when 2D nonlinearities were sampled over 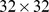 domain, and in general dropped due to overfitting when 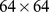 bins were used. We used 2D LN models fit on the nonrepeated segment of the stimulus to predict the PSTH for the repeated segments, using the time resolution of 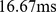, half the stimulus refresh rate. The fit was quantified by computing the Pearson cross-correlation between the true and predicted PSTH.

### Information captured by the models

In the framework of LN encoding models, one assumes that only a small number 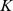 of linear projections 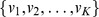 of a high-dimensional stimulus 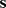 determine whether a neuron spikes or not [41]. In other words, the neuron is viewed as implementing a probabilistic dependency chain: 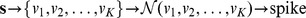, which implies a chain of information processing inequalities: 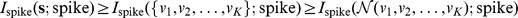. It is possible to estimate 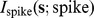 from repeated presentations of the same stimulus. If 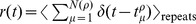 is the time-dependent firing rate, where 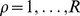 indexes the repeats, 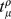 is the time of 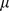-th spike in repeat 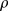, 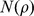 is the total number of spikes in repeat 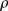, and 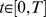 denotes time within the repeat of length 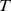, the estimate for true information per spike is given by [21]: 
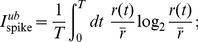
(2)here 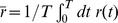 is the average firing rate across the repeated stimulus segment. This quantity is an upper bound to the information quantities defined above for LN models. The fraction, e.g. 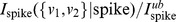 (between 0 and 1), tells us how well the two stimulus projections capture the full dependence of spiking on the stimulus. Similarly, 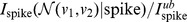 (which needs to be lower or equal to the information in the two projections for the same neuron and stimulus) quantifies how much information is further lost when compressing the description of spike-dependence from two projections into a single nonlinear combination.

The information was estimated from Eq (2) with rates computed in 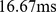 bins (matching the time resolution of the temporal filters and the sampling used to calculate 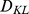 in Eq (1)), and was corrected for small-sample bias by repeatedly estimating the information on random subsets of stimulus repeats of varying sizes, plotting the information estimates against 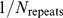 and extrapolating to infinite number of repeats; we also applied the correction for the difference between mean firing rates in the repeated and non-repeated stimulus segments [42]. The expected extrapolation error is below 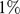. To obtain an upper bound for the systematic error due to short repeat length, we split the repeated segment in half and estimated the information separately on each half, which resulted in relative differences with a std of 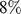; we expect the true error to be smaller. Information rates were estimated by computing the information per spike and multiplying by the mean firing rate.

For all neurons recorded in experiment 2 (with non-repeated and repeated stimuli), we computed several information-theoretic quantities: (i+ii) 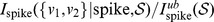 is the information fraction captured by 2 (and 1, respectively) linear filter(s), fit *separately* to each stimulus condition 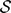; (iii+iv) 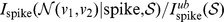 are the fractions captured by the nonlinear combination of 2 (and 1, respectively) projection(s) fit *separately* to each stimulus condition 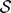; (v+vi) 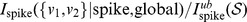 and 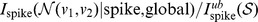 are the fractions captured by a single 2D model (by two projections and their nonlinear combination, respectively) that has been fit *globally* to all stimulus stimuli 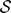. These quantities were all estimated using CTW estimator for Kullback-Leibler divergence. When estimated on spike trains that have been shuffled with respect to the stimulus, the estimator yields negligible values below 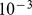 bits.

### Optimizing the filter shape for information transmission

The biphasic filter was parametrized by two parameters 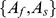 in a raised cosine-bump basis, where the basis was given by 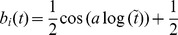, for 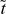 such that 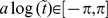, and zero elsewhere; 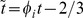, where 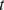 is the time measured in 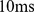 stimulus frames in the simulation. A similar basis has been used before for modeling the temporal filters in the RGCs [43]. For our two filters we used 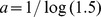; 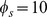 and 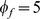 specify the peak time and the width of the slow and fast filters, respectively.

### Predicted information rate of LN models

To computationally simulate the effect that the (lack of) adaptation would have on the information rate when the stimulus statistics changes, we used the LN models inferred at high contrast C++ to predict the firing rate 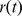 for stimuli with contrasts C
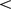
C++ and ask how much information such neurons would carry per spike in the absence of any adaptation. This information in the predicted rate, 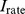, was evaluated using Eq (2) and expressed as a fraction of information captured by the two relevant filters (which does not depend on contrast and only serves as a normalization). We similarly asked how the same quantity would behave when the global models (single LN models for all cells that are fit across all stimuli, and therefore have no adaptation) were used to encode information into the rate for each of the skewed stimuli.

## Results

**Figure 1:**
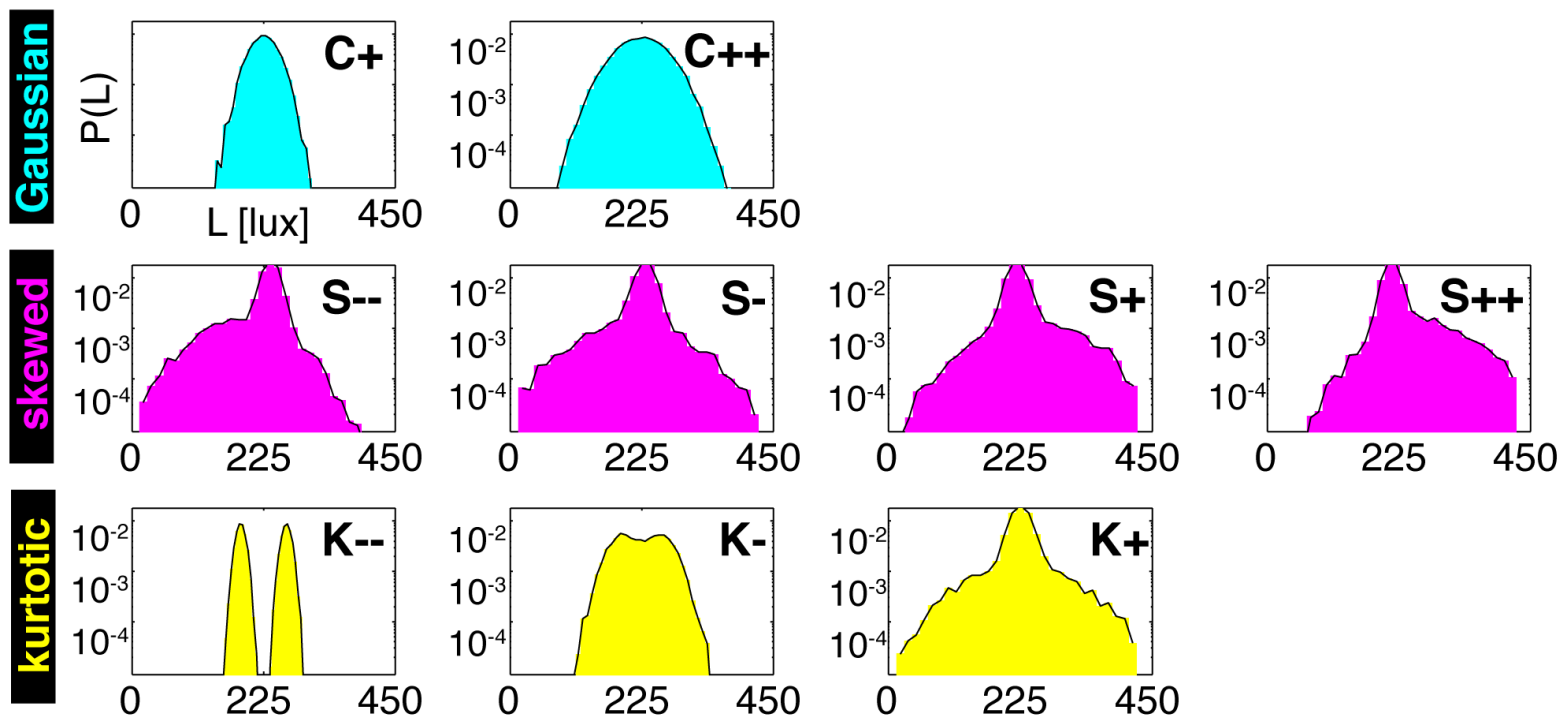
Synthetic stimuli used to probe salamander retinal ganglion cells. The stimuli are spatially uniform with light intensity 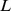 drawn independently on each stimulus frame from 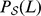. The probability densities, 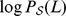, for all 9 stimuli 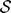 used, grouped into 3 categories (cyan  =  Gaussian, magenta  =  skewed, and yellow  =  kurtotic). All stimuli are matched in mean (225 lux), and all except for C+ have the same contrast; for details, see Table 1.

**Figure 2:**
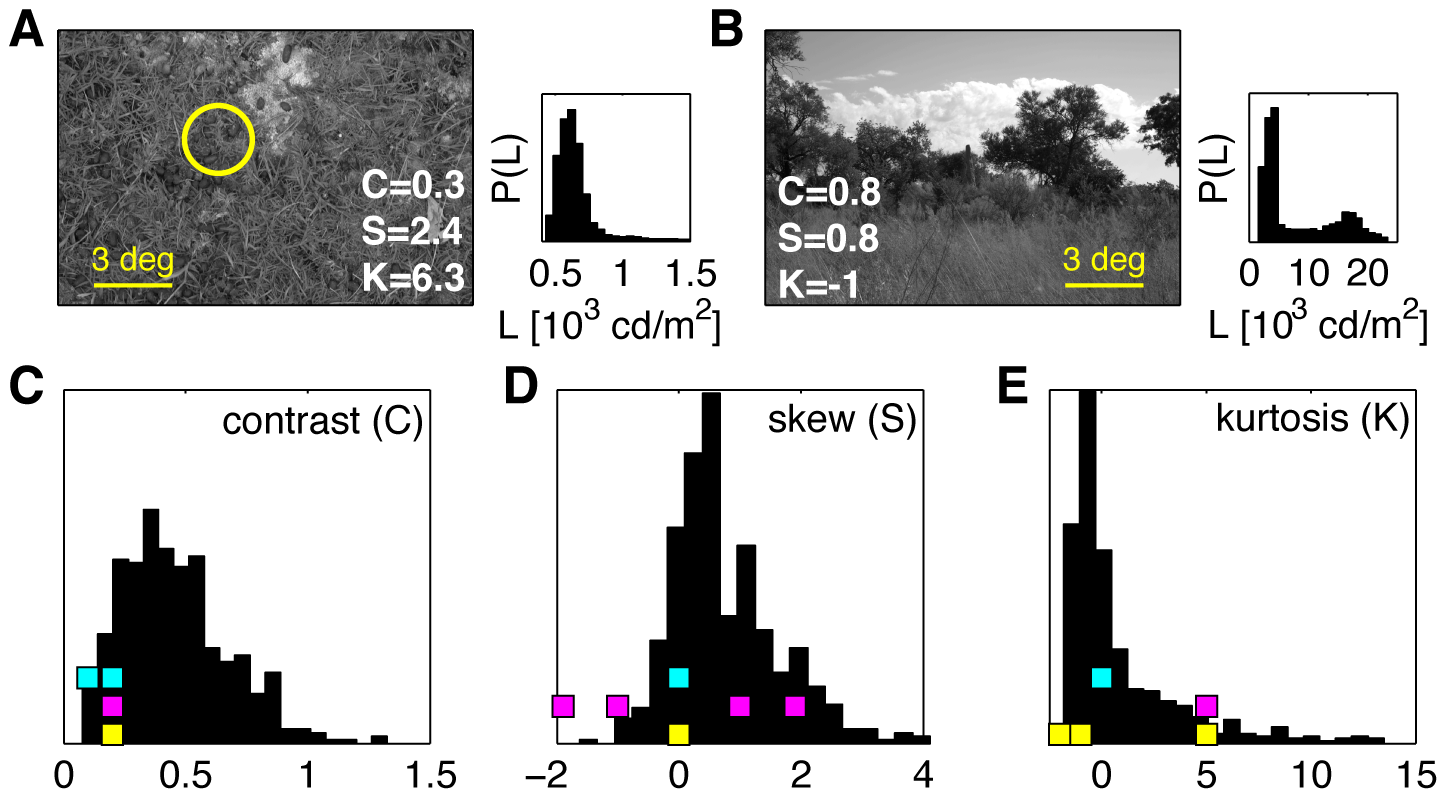
Higher-order statistics in natural scenes. **A,B**) Two example images from the Penn Natural Image Database [32]. The grayscale images are calibrated into units of cd/m^2^. The yellow circle represents the typical size of the salamander retinal ganglion cell center. Luminance was averaged in patches of this size, and contrast (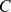), skewness (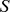) and kurtosis (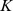) were computed for the distribution over many patches from each image. The distributions 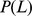 for the two example images are shown as insets, and the corresponding values for 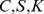 are displayed in the two image panels. **C,D,E**) The distribution of contrast, skewness and kurtosis, respectively, over 501 natural images. Colored squares represent the values of the three parameters used in synthetic stimuli (color coded as in Fig 1). 2 cyan stimuli differ in contrast 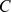 but have constant 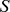 and 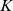; 4 magenta stimuli differ in skew 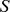 but have constant values of 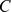 and 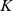; and 3 yellow stimuli differ in kurtosis 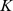 but have constant 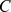 and 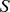 (see Table 1).

To characterize how the retina encodes higher-order statistics (HOS) of the luminance distribution, we presented it with a set of 9 synthetic spatially homogenous stimuli 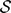, where the light intensity of each stimulus frame was drawn independently from distributions 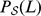 that were matched in mean 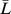 (see Materials and Methods; Table 1). The stimuli differed systematically in contrast, skewness, and kurtosis, as depicted in Fig. 1. To find the relevant range over which to vary these parameters in our synthetic stimuli (given that we could only make stable recordings with 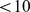 different stimuli on a single retina), we analyzed a set of calibrated natural images and extracted the histograms of contrast, skewness and kurtosis of light intensity. Based on this analysis, we picked 4 different values for skewness, and 3 different values for kurtosis, in addition to non-kurtotic non-skewed Gaussian distributions, as shown in Fig. 2A–E. Note that the range of statistics selected in this way is much broader, by a factor of up to 5, than what was used previously in a related study of Ref [31]. To span these ranges for skewness and kurtosis, contrast values 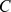 had to be chosen in the low range, due to the hardware limitations of the stimulus display. We generated HOS luminance distributions as mixtures of Gaussians. Gaussian mixtures represent fast and easy-to-implement parametrizations for the stimulus distributions, which can easily be reproduced with the parameters given in Table 2.

To quantify how retinal neurons change their code when contrast, skewness, or kurtosis of the stimulus change, we constructed accurate encoding models for the recorded neurons, and compared their properties under the different stimuli. We thus followed Ref [42], who have shown that for spatially uniform Gaussian stimuli in the salamander retina, linear-nonlinear (LN) models with one or two linear filters often suffice to describe the cells' encoding scheme with high accuracy. Moreover, Ref [40] also provided an interpretation of the filtering operations as dimensionality reduction on the stimulus space, the success of which can be quantified with information theory. Here we extended their framework to non-Gaussian stimuli and analyzed how information is encoded beyond the linear filtering stage, in the nonlinear response, and finally in the spiking rate. We could then characterize adaptation quantitatively, and compare the behavior of real neurons with computational models that either have or lack adaptation.

### Linear filters of retinal ganglion cells responding to HOS stimuli

**Figure 3:**
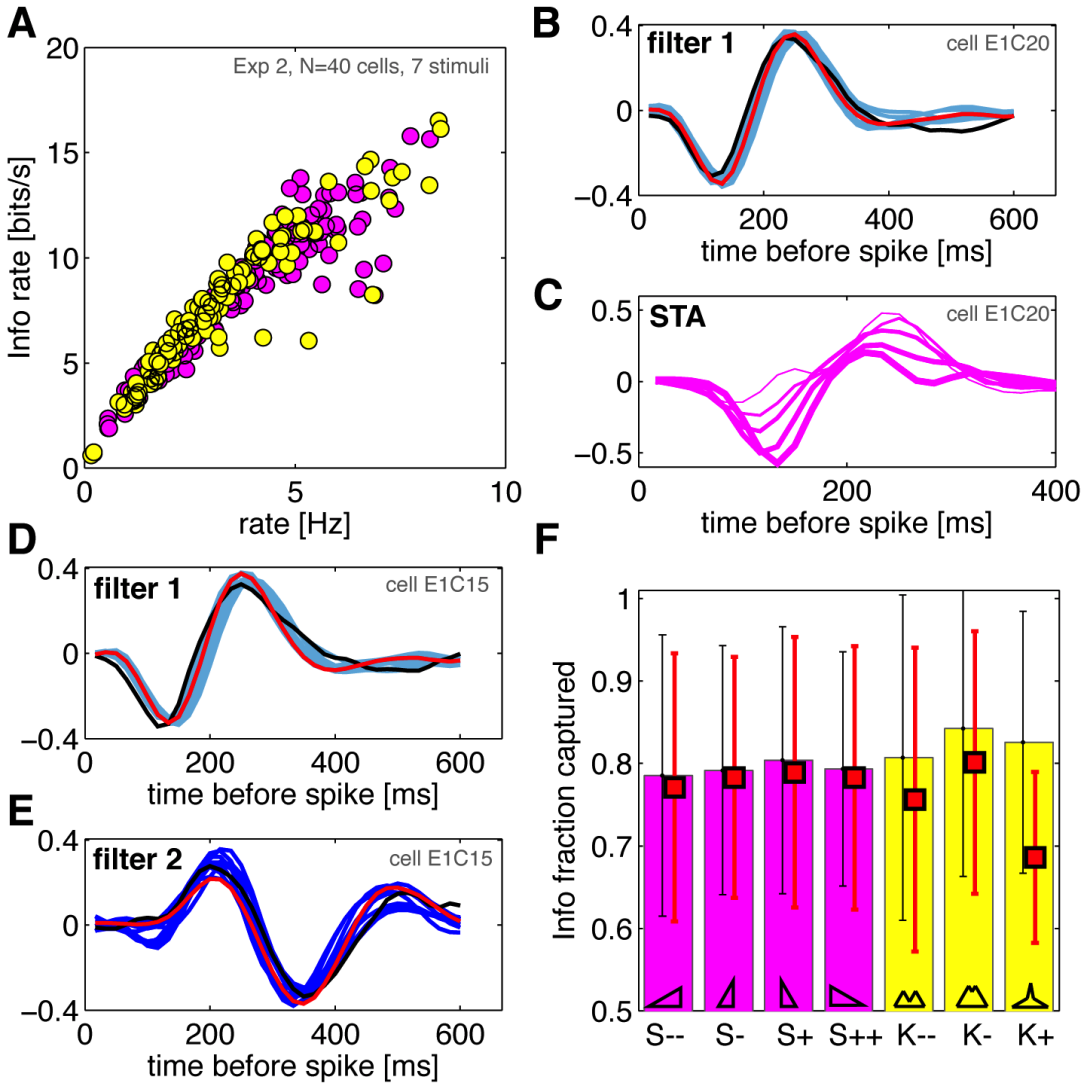
Linear filters and higher-order statistics stimuli in retinal ganglion cells. **A**) Estimated information rate (see Methods) as a function of the mean firing rate. Each dot represents one of the 40 cells in experiment 2 exposed to one of the 7 HOS conditions (skewed stimuli in magenta, kurtotic in yellow). The growth in information is slightly sublinear, with no obvious systematic dependence on the stimulus type. **B**) A cell whose behavior is captured well by a single linear filter. Shown in light blue are the filters for all 9 (2 Gaussian, 7 HOS) stimuli reconstructed using maximally informative dimensions; in black the spike-triggered average computed on the Gaussian stimulus C++; in red, a single *global* filter inferred using MID across all stimulus conditions simultaneously. **C**) Biased STA filter estimates for 5 skewed stimuli (thicker lines mean increasing skewness) for the same cell as in B (note the difference in the time axis). **D,E**) A cell whose behavior is described well by two linear filters (light blue  =  the most informative dimension; dark blue  =  the second most-informative dimension). Other symbols the same as in B). **F**) Information captured by two filters (across stimuli, horizontal axis), as a fraction of the total information per spike; mean (bars) and interquantile range error bars across 40 cells in experiment 2. The average performance of global models (the same pair of filters across all stimuli for each cell) is plotted as red squares.

We recorded from 23 retinal ganglion cells that were presented with 9 types of non-repeated stimuli 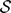 (2 Gaussian + 7 higher-order statistics, Fig. 1) in experiment 1, and from 40 cells presented with non-repeated and repeated stimuli of 7 types with higher-order statistics in experiment 2 (see Materials and Methods). Figure 3A shows the estimated information rate of the neurons as a function of their firing rate. Consistent with previous reports [44], the information rate 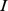 scaled weakly sub-linearly with the mean firing rate 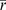 (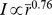). There were no other large systematic dependencies in transmitted information across cells and stimulus classes.

Next, we inferred the best linear filters for each cell, and each of the stimulus conditions 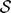, separately. We used maximally informative dimensions (MID) for learning the filters for all stimuli [37], and for the Gaussian stimuli we additionally used spike-triggered average and spike-triggered covariance. We also inferred a *global* model for each cell, where a single set of filters was fit across all stimulus conditions (see Materials and Methods). In cross-validation on test data, the prediction performance of the models of essentially all cells (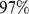 of cell/stimulus combinations) increased when using two filters (2D LN models), compared to one-dimensional LN models, but in some cases the contribution of the second filter was very small. The linear filters inferred using MID for one of these cells are shown in Fig. 3B for 9 stimulus conditions; overlaid is the leading eigenvector of the spike-triggered covariance matrix computed for the C++ stimulus, and the best global filter learned by MID. All filters are scaled to unit norm. The filters show very strong overlap, indicating that their shape does not adapt substantially to the stimulus distribution. We emphasize that computing the naive spike-triggered average (STA) estimates gives a systematic change in filter shape with the stimulus skew, as shown in Fig. 3C, but this is simply an artifact of the STA estimation on non-spherically-symmetric stimuli [38], [45], [46], and is not indicative of any adaptation process.

Figures 3D–E show a typical cell for which a model with two linear filters is needed. We again observe a high overlap between the filters inferred using MID in 9 stimulus conditions, the filter pair computed using spike-triggered covariance (STC) in the Gaussian condition, and the single global best pair of filters inferred across all conditions using MID. 15 of the 23 cells in experiment 1 have an average firing rate above 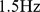 for every stimulus, permitting reliable filter estimation. Out of those, a single-filter model in the Gaussian condition suffices for 8 cells, i.e., the single-filter model accounts for more than 90% of the information per spike of the two-filter model. For 7 cells two filters are needed. To measure the agreement between inferred filters across conditions for each cell, we compute the overlap (scalar product; the filters are unit Euclidean norm) between the STC derived filter(s) in the Gaussian condition, and the filters derived using MID for each stimulus condition 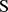. The average overlap across the group of cells with a single linear filter is 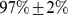, while the average overlap across the group of cells with 2 filters is 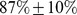 (error bar  =  std across cells). The decrease in the latter case is most likely attributable to the difficulty of inferring jointly 2 filters using MID with a limited number of spikes; we observe a systematic decrease in correlation for smaller numbers of spikes, and the estimates of the second filter are noticeably more noisy than the estimates of a single (first) filter.

**Figure 4:**
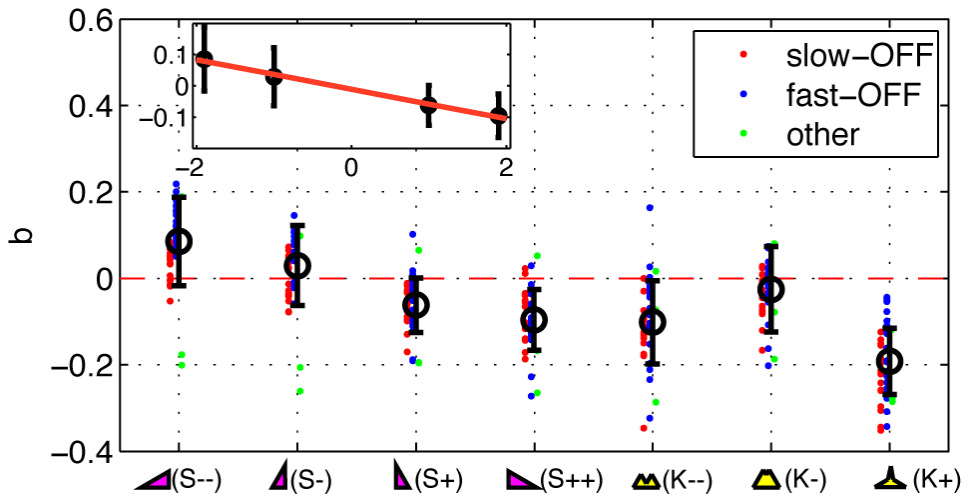
The dependence of the balance index on the stimulus type. The balance index 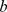 is a ratio between the total (signed) area under the most significant linear filter of each cell, normalized by the absolute area of the filter; balanced filters have 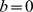, fully unbalanced 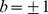. Individual dots represent 40 individual cells from experiment 2, which have been grouped into fast-OFF and slow-OFF classes (blue and red, respectively), and a small group of unclassified cells (green). Black symbols show the averages (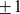 std error bars across the recorded population). Inset shows the dependence of the balance index 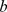 as a function of skewness 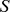 drawn to scale; the best linear fit (red) is 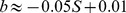.

**Figure 5:**
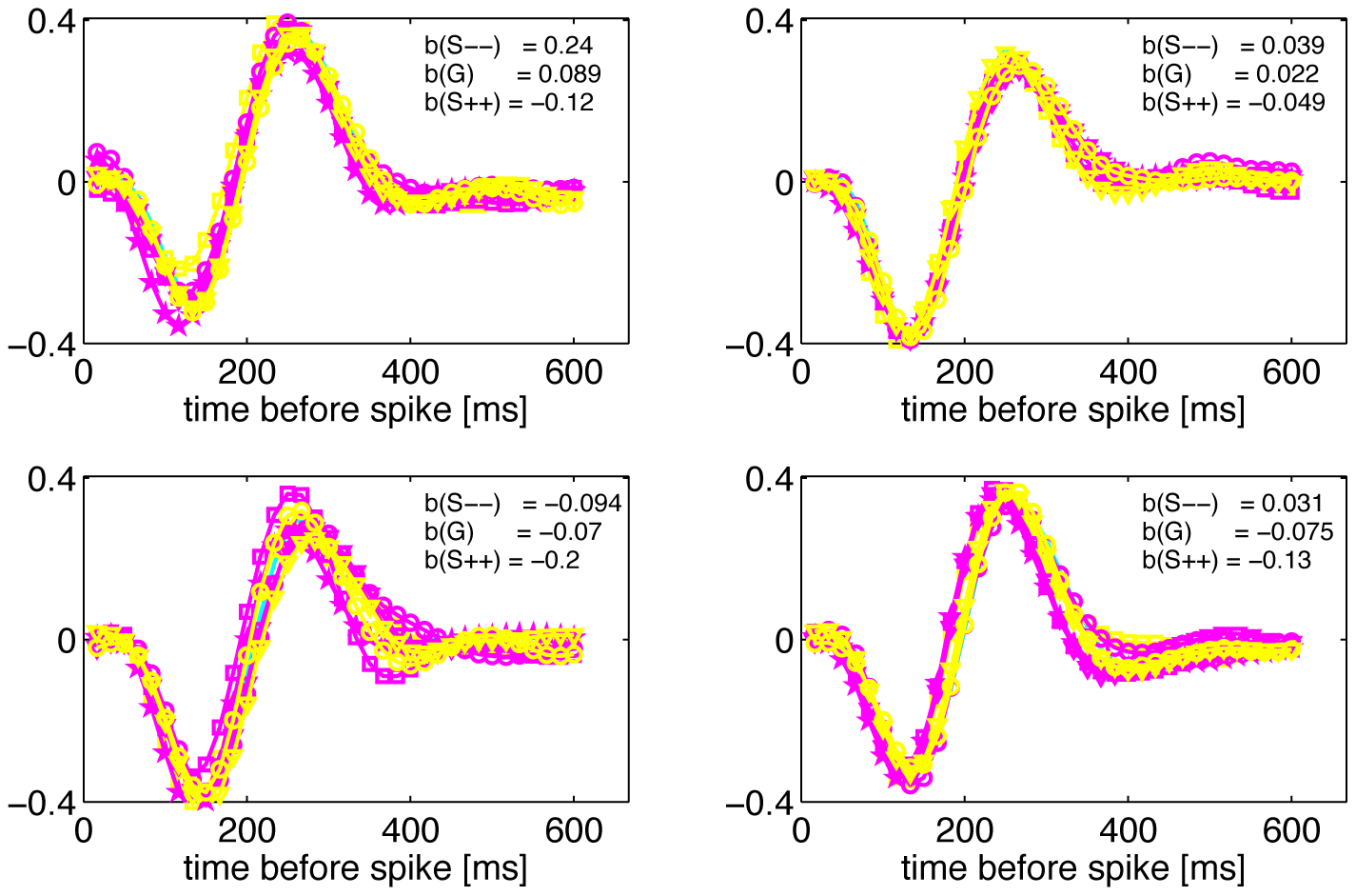
Linear filters and balance index for four example cells in different stimulus conditions. Each panel shows the linear filter in four skewed conditions (magenta), three kurtotic conditions (yellow), and the gaussian condition of matched variance (cyan). 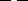 stimuli are denoted by circles, 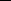 stimuli by triangles, 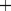 stimuli by squares, and 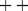 stimuli by stars. The balance index for 3 selected filters (two extreme skewed distributions and the zero skew condition, G) is reported in each panel.

We quantified the effects of changing the higher-order statistics on the shape of the linear filters by computing the balance index 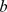. 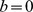 indicates a balanced filter that yields zero output on a temporally constant signal, thus behaving as a differentiator, while 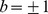 indicates a completely unbalanced filter that behaves as an integrator. More precisely, 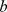 is defined as the ratio between the total (signed) area under the filter and the absolute area: 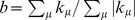, where 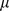 indexes the temporal components of the filter. The balance index across the recorded population was 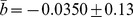 (n = 40 cells from experiment 2), where the mean and error bar (std) are taken across all cells and all conditions. Broken down across conditions, there is a small systematic modulation of 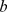 with the stimulus (Fig. 4 and inset), with a std of about 0.09 (see Methods for an error estimate for our determination of 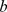; we use a conservative estimate of 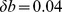 due to stochastic optimization and finite number of spikes). This variation in median value of 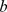, while fractionally small, is statistically significant at 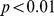 between 16 out of 21 pairs of conditions, amongst others, between S−−, S−, and all other conditions, and between K+ and every other condition but S++, between some other pairs. Significance was assessed using bootstrap resampling to estimate the distribution of the median difference between pairs of conditions, assuming IID gaussian errors of magnitude 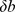 for every cell, and testing against the null hypothesis that the difference is consistent with zero; significance test was Bonferroni corrected for multiple comparisons. We have also clustered the neurons into two major classes, fast-OFF and slow-OFF (along with a few unclassified cells; in salamander retina, OFF-type cells account for 80% of all retinal ganglion cells as reported in Ref [47]); for two left-most skewed stimuli, S−− and S−, cells in the two classes have significantly different mean balance index (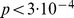, two-sided t-test), while for the other stimuli the differences are not significant. We provide further examples of the most significant linear filter in each condition for 4 more cells in Fig. 5.

To ask whether these slight variations in filter shape across stimuli matter for encoding, we compared the performance of global filters, constant for each cell across all the stimuli, with filters inferred separately for each stimulus. We estimated the information captured by the single-filter model, by a two-filter model, and by a two-filter global model, where a single pair of two filters is inferred for all stimuli for each cell. Single-filter models (fit to each stimulus separately) captured 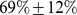 (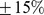 interquartile-range, or IQR, which measures the spread around the median that contains 50% of the data) of the information per spike (averaged across cells and stimuli). Two-filter models (fit to each stimulus separately) captured 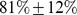 (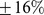 IQR) of information per spike, as shown in Fig. 3F; for some cells, two filters capture essentially all of the information. Our observations were quantitatively consistent with the results reported previously [42].

We compared this information capture with the performance of global models, where the filters for each cell do not change with the stimulus condition. Averaged across conditions, the global models captured 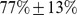 (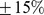 IQR) of the information per spike. Information captured by separate models *must* be higher or equal to the information captured by the joint model, by construction; we next asked about the significance and magnitude of this difference. In all skewed conditions (S−−, S−, S+, S++), the median differences were small (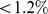), while for K−−, K−, and K+, the differences were larger, 4.9%, 3.9%, and 13%, respectively. For all skewed conditions, the information captured by separately inferred filters was not significantly different from the information captured by the global filters, while it was different for kurtotic conditions (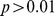; significance was assessed using bootstrap resampling to estimate the distribution of the median difference between separate and joint capture, and comparing to null hypothesis of zero difference; we assumed conservative IID gaussian errors of 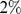 on information capture for every cell; see Methods).

Our results show fractionally small changes in the shape of the linear filters for salamander retinal ganglion cells in response to changes in skewness and kurtosis. For changes in skewness, these observed changes in the filter shape change the amount of information per spike captured by an amount that is close to the resolution of our inference method; for changes in kurtosis, the changes are larger.

### Nonlinearities of retinal ganglion cells responding to HOS stimuli

**Figure 6:**
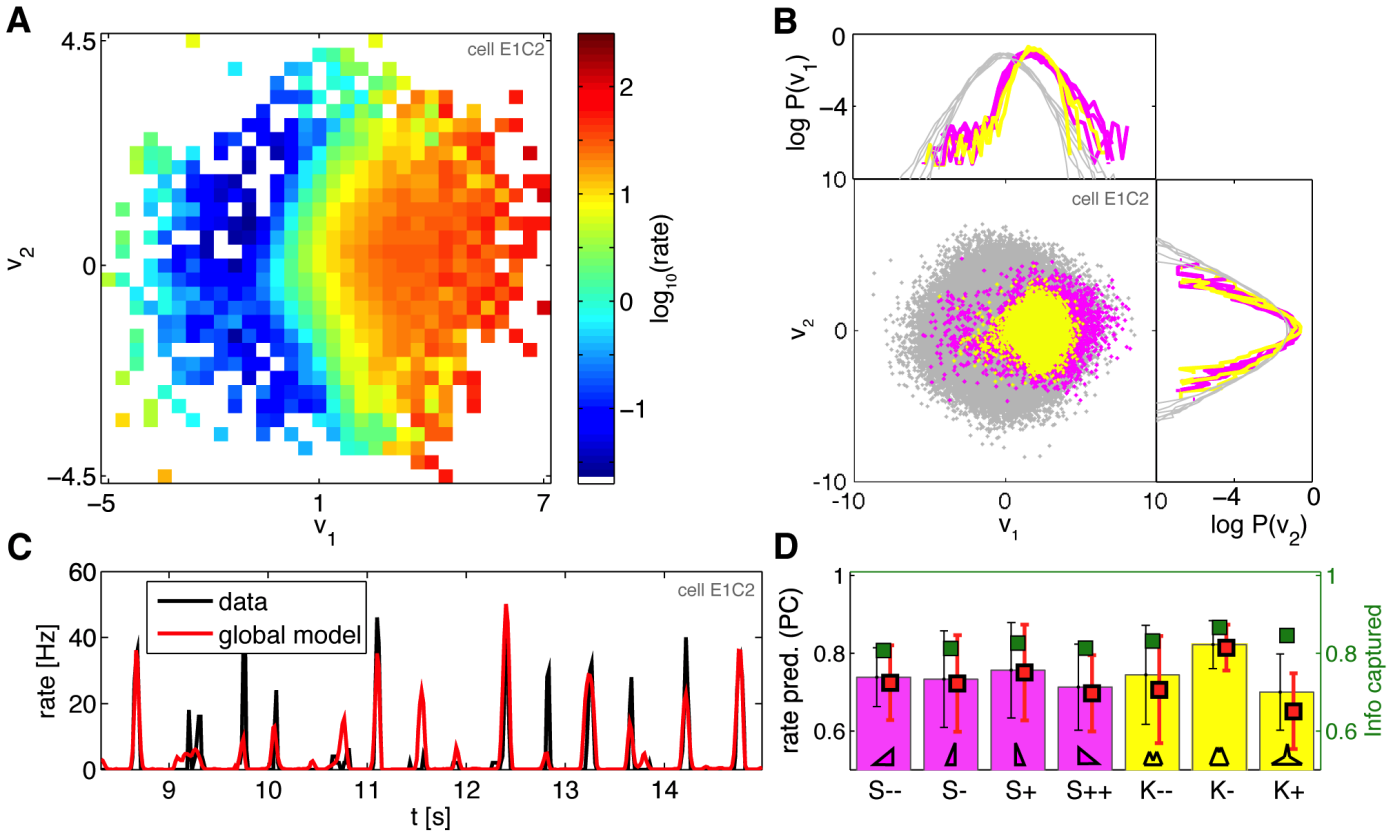
Nonlinearities and rate prediction with higher-order statistics stimuli. **A**) A 2D nonlinearity globally fit across all HOS stimuli for neuron E1C2; projection of the first (second) filter shown on the horizontal (vertical) axis. Hotter colors indicate increased firing rates (see colorbar, rate in Hz; white  =  regions of 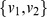 space where no spike or prior samples have been observed). **B**) For the same cell, the depiction of prior ensemble (gray dots, all 7 higher-order statistics stimuli overlaid) and the spike-triggered ensembles (magenta  =  skewed stimuli, yellow  =  kurtotic stimuli); shown are also projections of the data, i.e. the marginal distributions 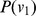 and 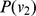, on the logarithmic scale, for all 7 stimuli separately. **C**) The segment of predicted and true firing rate in responses to repeated K− stimulus presentations (red  =  2D LN global model fit to all stimuli for this neuron; black  =  true rate). **D**) Model performance, measured as the Pearson correlation (PC) between the true and predicted PSTH, across different stimuli (horizontal axis; average and error bars  =  mean and interquartile range across 40 neurons in experiment 2). The performance of 2D LN models fit separately for each stimulus is shown by magenta (skewed stimuli) and yellow (kurtotic stimuli) bars. Global model performance (red squares) matches the performance of models fit separately. Right axis, in green: information fraction captured by the nonlinear combination of the 2 linear projections, 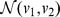, shows no drop compared to the information captured by the linear features themselves (c.f. bars in Fig. 3F), and is between 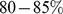 across all stimuli (error bars omitted for clarity, comparable to error bars in information fraction captured by the 2 linear features).

Completing the LN description of the ganglion cells is the mapping from the linear projection(s) of the stimulus into the cell's firing rate. We estimated these 2D nonlinear functions from the data by binning 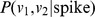, where 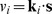 are the projections of the stimulus onto the two filters, 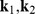, as explained in Materials and Methods. This was done for each neuron and each condition separately, or for all conditions jointly using the *global* pair of filters, to yield a single 2D global LN model for every cell. Figure 6A shows a global nonlinear function for an example neuron. In Fig. 6B we explicitly show, for that same neuron, the prior ensembles for all 7 higher-order statistics stimuli (gray), with overlaid spike-triggered ensembles for skewed (magenta) and kurtotic (yellow) stimuli, along with the marginal projections of these distributions. We first estimated how much information is lost in compressing the 2D projections 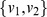 into the nonlinear combination, 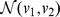, on average. As shown in Fig. 6D, the nonlinearity captured 80–85% of the information per spike (fit for each condition separately), essentially the same amount as the two linear filters (c.f. Fig. 3F); mathematically, this means that 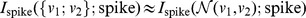 (see Methods). This finding establishes that the nonlinear mapping itself does not discard the information per spike, and that analyzing the changes in point-wise nonlinearities is warranted.

Does the nonlinearity change with the stimulus condition? The number of spikes is in general insufficient to reliably sample and then systematically compare the 2D nonlinear functions across cells and stimuli. We thus decided to base our comparisons directly on the firing rate prediction performance. The 2D models for cells in experiment 2 are fit on the non-repeated segments, and are subsequently tested by predicting PSTH in response to repeated stimulus presentations for which we could measure the true PSTH; here, too, the prediction was done either with models fit separately at each condition, or with the global model, where 2 linear filters and the nonlinearity were fit simultaneously across all stimuli, as shown in Fig. 6C. In terms of PSTH prediction, the prediction of the 1D LN models, fitted to each conditions separately, had 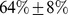 (9% IQR) correlation with the real PSTH (error bar  =  std across 40 cells and 7 stimuli). 2D LN models were better with 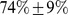 (10% IQR), as shown in Fig. 6D. The global models that had constant filters and nonlinearity across 7 higher-order statistics conditions, performed negligibly lower, with 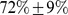 (11% IQR) correlation. Condition by condition, the differences between global and separate models were small (median differences of 1.1%, 1.3%, 0.2%, 1.9%, 3.2%, 1.1%, 5.8%, for S−−, S−, S+, S++, K−−, K−, K+, respectively) and statistically insignificant for all conditions except K−− and K+ (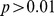; significance was assessed using bootstrap resampling to estimate the distribution of the median difference between global and separate models and comparing to zero difference null hypothesis; included was an estimated gaussian IID 3% error on PSTH prediction due to a limited number of stimulus repeats for computing the true PSTH). Similar to the comparison of linear filters, the largest difference is observed at K+ condition; this is also the condition where the spike rate is lowest and the models are hardest to infer.

**Figure 7:**
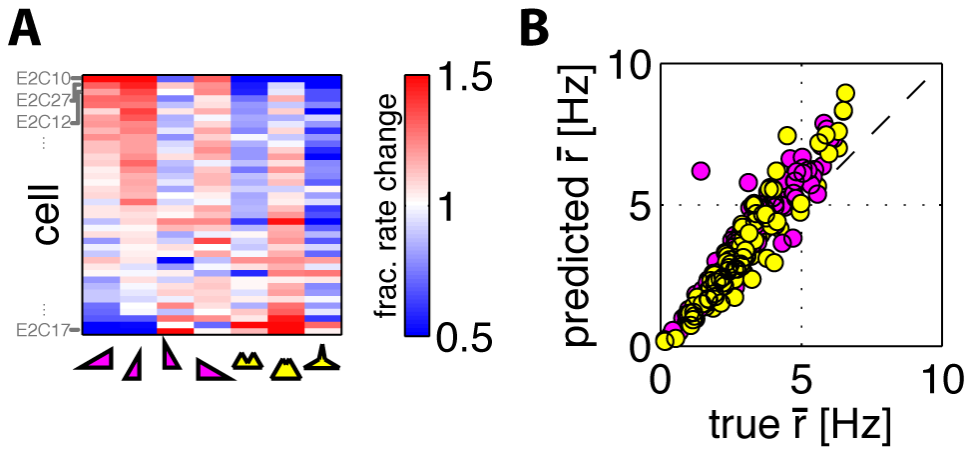
Measurement and prediction of changes in the mean firing rate with stimulus condition. **A**) The relative change (color scale, 1  =  the firing rate of the cell is equal to the mean rate for that cell across all stimuli) in the mean firing rate for 40 cells of experiment 2, as a function of the stimulus condition (magenta  =  4 skewed, yellow  =  3 kurtotic stimuli). Neurons (rows) were sorted by the projection on the first principal component explaining most of the change across the recorded population; cells close to the top increase the firing rate in response to left-skewed stimuli, while cells at the bottom increase the rate in response to right-skewed and negative kurtosis stimuli. **B**) Global 2D models for each cell predict the average rate well (each dot is one cell in one of the 7 HOS stimulus conditions).

Finally, we can ask how successfully the global models recapitulate the overall firing rate changes with the stimulus statistics. Figure 7A shows the relative change in firing rates for cells from experiment 2 for 7 HOS stimuli; the cells have been sorted to reveal the dominant pattern, where cells that prefer left-skewed stimuli respond less strongly to the other stimuli, while cells that respond strongly to right-skewed stimuli also respond to negative kurtosis. We can use a global 2D LN model fit for every cell to predict separately the mean firing rate in response to each of the 7 stimuli; note that the model is only constrained to fit the overall firing rate (across all conditions together). These models that lack any adaptation nevertheless reproduced very well the mean firing rate for each stimulus and cell, as depicted in Fig. 7B, and therefore also the pattern of changes in the firing rate.

### Comparing the observed HOS-induced effects to contrast adaptation

We observed that the encoding properties of salamander ganglion cells depend on higher-order statistics of the luminance levels in a way that is statistically significant for some of the conditions, but the observed changes in model parameters and the related model performance measures were fractionally small, usually ranging from no significant change to 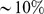. Here we ask whether these changes are large or small in comparison to those elicited by the well-characterized contrast adaptation mechanism, and whether there is any theoretical reason for adaptation to contrast to be different from the adaptation to higher-order statistics.

**Figure 8:**
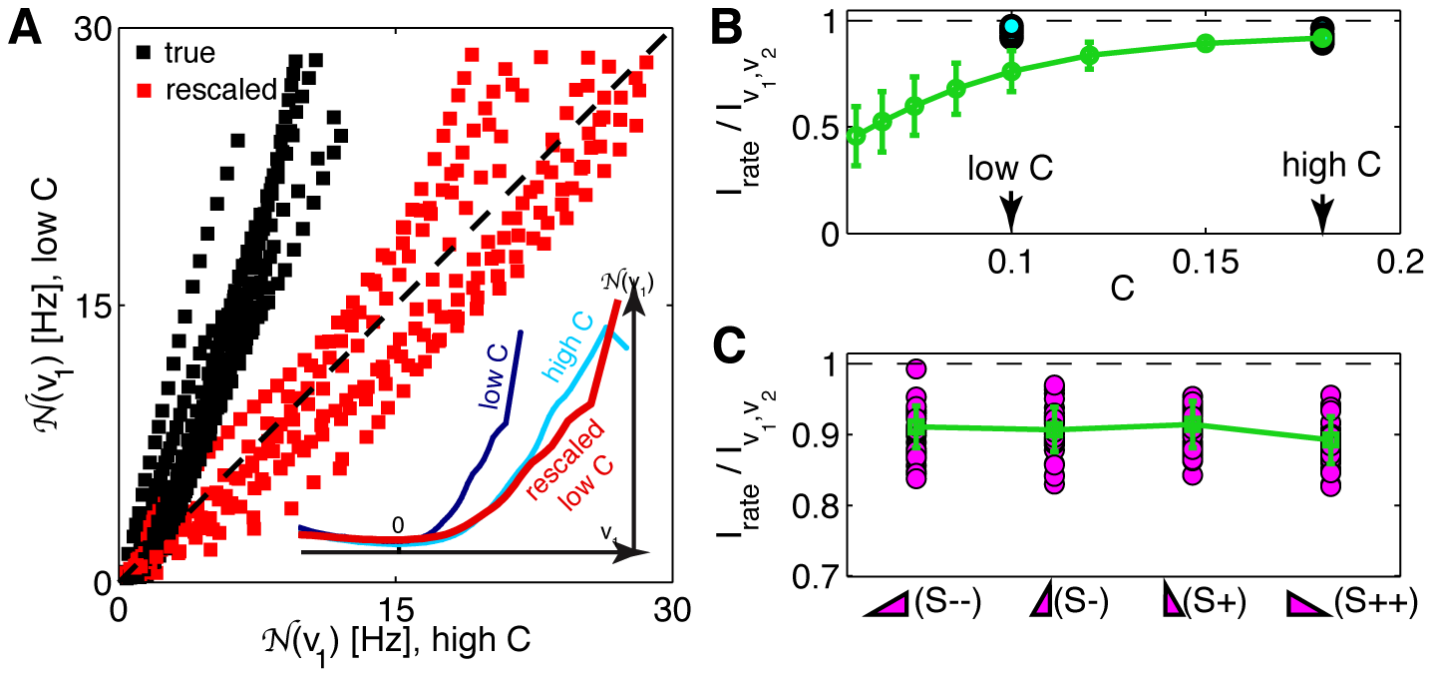
The benefits of contrast and higher-order statistics adaptation. **A**) *Inset.* The 1D nonlinearity, 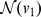, for an example neuron (E0C4) inferred at high contrast (C++, light blue), and at low contrast (C+, dark blue). The low contrast nonlinearity can be aligned to the high contrast one by (i) rescaling the stimulus (horizontal) axis by the ratio of the two contrasts, and (ii) rescaling the firing rate (vertical) axis by the ratio of the two average firing rates, yielding the red line. *Main panel.* Scatter plot of the nonlinearity at high vs nonlinearity at low contrast (black, 19 neurons from experiment 1; the coordinates of each point are the high/low C nonlinearity values at the same value of the projection 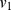 for a particular neuron). After rescaling, the nonlinearities align (red). The scaling breaks down for rates above 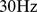 (rarely observed at low contrast). **B**) The information in the spiking pattern of a LN model neuron, normalized by the information captured by the two linear projections of the corresponding stimulus. Cyan circles  =  inferred models for 19 neurons for high and low contrast (C++, C+) stimulus. Green line  =  computational prediction obtained by taking 19 high contrast models and dialing down the stimulus contrast without any adaptation in the model (error bars  =  std across the neurons). **C**) Analogous analysis for changes in skewness (note the difference in scale); magenta  =  models inferred separately for each skewed stimulus; green  =  invariant (and therefore non-adapting) global models for every cell.

We analyzed the recordings from experiment 1 where neurons were also exposed to high (C++) and low (C+) contrast Gaussian stimuli. Since our analysis kept the filters normalized to unit length, contrast adaptation would be reflected in the change of the shape of the nonlinearity. Indeed, this can be seen in Fig. 8A (inset), which shows the (marginal) nonlinearity, 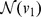, of a typical neuron for the high- and low-contrast experiments. We then took the nonlinearity from the low-contrast experiment and rescaled it as follows. First, we rescaled the range of inputs to the nonlinearity, 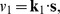 by the ratio of high to low contrast, C++/C+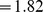. Second, we also rescaled the output firing rate by the ratios of the steady-state firing rates in both C+ and C++ conditions (the rates are not equal because the neurons do not adapt perfectly). After these two rescaling operations, the *measured* nonlinearity for C++ (high contrast) stimulus lined up well with the *rescaled* nonlinearity from the C+ (low contrast) stimulus, indicating the ability of the neuron to adapt to contrast. This observation was generally true for most (19 out of 23) neurons in our dataset, as shown in Fig. 8A. The rescaling fails at very high firing rates, because they are not accessed in the low contrast condition, and (potentially) because we were only looking at the marginal 1D (and not the full 2D) nonlinearity. Importantly, the nonlinearity gain during contrast adaptation changes by the same factor as the contrast—in this case almost two-fold—implying that changes in contrast experienced during natural vision will lead to changes in gain that are not just fractionally small as they appear for HOS stimuli, but can easily exceed 100% [13], [14], [19], [20], [21], [22].

When the stimulus contrast changes, retinal ganglion cells adjust their gain, matching the variation of the signal about the mean to the dynamic range of the firing rate at the output, thereby keeping the information rate high. Without adaptation, the information rate would drop because the neurons have a limited output range and they are noisy. “Noise” in the context of LN encoding models is the stochasticity related to the spike generation: from the stimulus 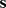 to spike, 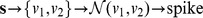, it arises when the nonlinear function 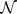 is interpreted as the mean firing rate of a Poisson point process. To computationally explore the effects of the presence or absence of adaptation, we generated spikes according to this LN prescription in response to various stimuli, and measured the information in such synthetic spike trains using Eq. (2). By using the true inferred (adapting) models for low and high contrast, we found that in both conditions the real spiking neuron can retain 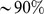 of the information extracted from the stimuli by the linear filters. On the other hand, when using the model inferred at high contrast, holding it fixed (no adaptation), and probing it with stimuli of progressively lower contrast, the information rate dropped significantly, as shown in Fig. 8B. The situation is very different for skewness (or kurtosis): Fig. 8C shows that no such drop in information is observed when the global model is used to generate spikes in case of skewed stimuli, making adaptation unnecessary and invariant encoding possible.

**Figure 9:**
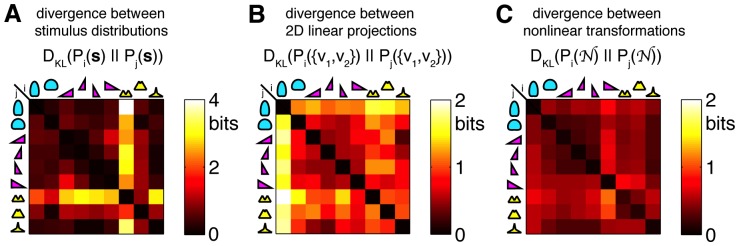
Neurons with contrast adaptation yield similar distributions of firing rates in response to very different distributions of inputs. Differences between distributions of stimuli before linear filtering, after filtering, and after nonlinear transformation, are quantified by Kullback-Leibler divergence matrix, 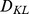, measured in bits [56]; while this is not a proper distance metric (since it is not symmetric), a value of 0 indicates identical distributions, and high values signify very different distributions. A) 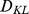 between all 9 pairs of stimulus distributions (cyan  = 2 Gaussian, magenta  = 4 skewed, yellow  = 3 kurtotic distributions). Bimodal K−− distribution is most different from the others. B) The difference between the respective 2D linear projections of the 9 stimulus distributions (shown are the averages over 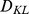 matrices for 19 neurons in experiment 1). Linear filtering of IID stimuli washes out most of the higher-order structure (but not the second order), and the most distinct stimulus type at this stage is C+, since its variance is different from the other distributions of projections. C) 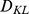 (average over 19 neurons) between the nonlinear transformations of the respective linear projections. Since the nonlinearity adapts to contrast, this step equalizes the low contrast (C+) with the other stimuli.

This effect is easy to understand if we compare the extent to which the 9 stimulus distributions differ a priori, after filtering by the neuron's linear filters, and after passing through the nonlinear function. For this analysis we used real linear filters and nonlinear functions reconstructed for all neurons in experiment 1 (see Fig. 9 caption for details). Figure 9A shows a 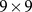 matrix of the Kullback-Leibler distances 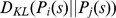 between all pairs of stimuli 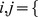
C+,C++,S−−,S−,S+,S++,K−−,K−,K+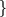 (2 Gaussian, 7 higher-order statistics). The bimodal stimulus K−− is clearly distinct from the others. After linear filtering (Fig. 9B), however, the low contrast stimulus C+ differs the most from the others, which are all mutually matched in contrast. Because linear filters, as we have shown, stay essentially unchanged in shape, this is simply a consequence of the central limit theorem: the filters sum up (with weights) samples drawn independently from the stimulus distributions 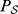, so the filter outputs must converge to Gaussian distributions with variances that are related to the variance (or contrast) of the input. In other words, the invariant linear filters remove the signatures of higher order statistics and “equalize” different stimuli with the exception of their contrast. In the last, nonlinear stage (Fig. 9C), the nonlinear functions adapt to contrast as well, ultimately yielding LN model outputs whose distributions are very similar across the range of stimuli differing in contrast, skewness and kurtosis.

In sum, the analyses of Figs. 8 and 9 suggest that contrast adaptation is qualitatively different from the putative adaptation to higher-order statistics. Without contrast adaptation, where the gain change must be of the same magnitude as the change in contrast itself, the information rate of the neuron would fall substantially (see also, e.g., [48]). A lack of adaptation to higher-order statistics does not lead to a drop in the observed information rate. The crucial role for this distinction is played by the initial, linear, stage, where higher-order statistics—but not contrast—are averaged away by the summation in the receptive field. Thus, so long as the linear stage can average over sufficiently large number of stimulus samples, efficient coding in LN models requires contrast adaptation, but not adaptation to higher order statistics.

### Optimal LN encoding of HOS stimuli

We have shown that the real neurons and their non-adapting model versions are essentially matched in the amount of information they can encode about the HOS stimuli. What remains unclear is how the real and non-adapting neurons are performing relative to *optimal* neurons, which could pick a separate linear filter for every stimulus condition so as to maximize the amount of encoded information. How much information gain, at best, could adaptation to higher-order statistics convey?

To answer this question, we considered a one-dimensional LN model neuron, whose probability of spiking was assumed to be a saturating nonlinear function of the filtered stimulus: 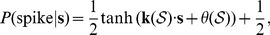
(3)where the linear filter 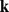 and the spiking threshold 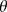 may depend on the stimulus type 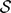. We simulated spike sequences 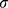 of this model neuron in response to repeated presentations of the stimulus, whose value in each time bin was drawn independently from from 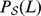: 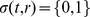 was ‘0’ when the neuron was silent in time bin 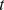 and during repeated presentation 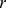 of the stimulus, and ‘1’ if it spiked. To quantify how well such a neuron encodes information about the stimulus into spike trains, we estimated the information rate 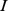 (in bits per second) between the stimuli and the response, using the direct method [49].

**Figure 10:**
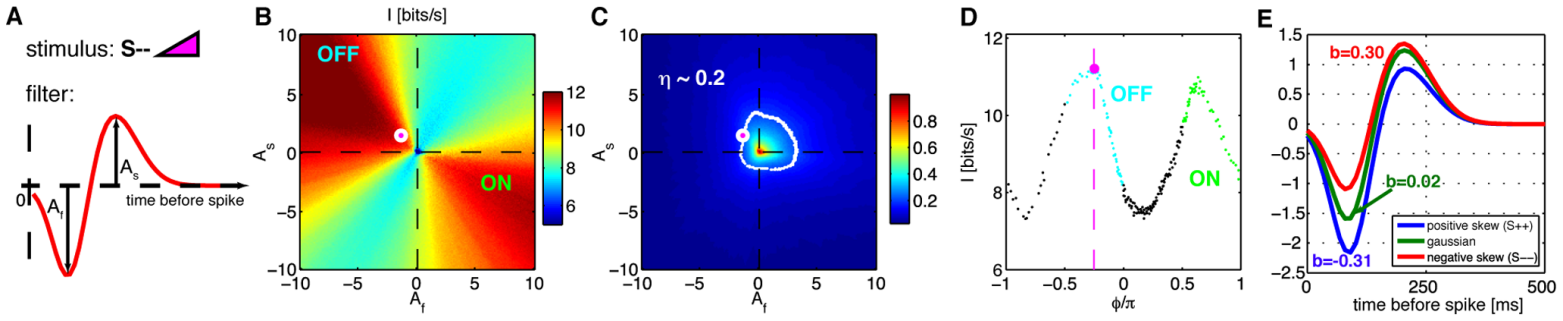
Finding optimal filters for an LN model neuron for stimuli with negative skew (S−−). **A**) The biphasic filter with two parameters determining the amplitudes of the fast and slow lobes, 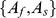. Each of the amplitudes can be positive or negative. When 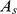 is positive and 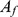 is negative, the cell is an OFF cell; when 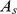 is negative and 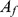 is positive, the cell is an ON cell. **B**) Information about the stimulus encoded in the spike train (bits per second, color scale), as a function of the fast lobe amplitude 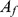 and the slow lobe amplitude 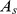. The ON and OFF types have been denoted in the 2 corresponding quadrants of the plot. **C**) Fraction of entropy lost to noise, 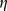, as a function of 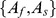. Points in the plane that have 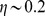 are shown in white, and lie on a circular locus of points; we are only interested in the models with fixed value of 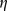. We parametrize such points by their angle, 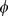, going counterclockwise from the vertical (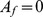). Magenta dot (here and in B, D) denotes the (OFF) filter that maximizes the transmitted information. **D**) Information on the locus of points 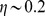 as a function of 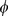; these values are extracted from B) along the 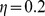 contour. Green points correspond to ON cells, cyan points to OFF cells. There are two peaks in information, one (slightly higher) peak for the OFF type cell and one for the ON type cell. For positive skew (S++, not shown for clarity), the results are analogous, with the maximum achieved for ON instead of OFF cells. **E**) Theoretical prediction for the shape of the optimal biphasic OFF filters for stimuli with different skewness values. As skewness increases from negative (S−−, red) to positive (S++, blue), the negative lobe becomes more prominent and the positive lobe becomes less prominent. For the symmetric Gaussian stimulus (C++, green) the optimal filter is balanced. These changes are quantified by the balance index 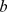 (see text), which measures the difference in area between the lobes, normalized to the total absolute area under the filter. For the simulations in this figure, the stimulus refresh time is 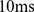 and mean firing rate is held fixed at 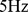 (results are qualitatively unchanged for rates up to fourfold higher).

For stimulus types 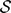 of varying skewness, we found the optimal filter 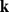 and the threshold 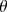 that would maximize the information rate 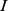 that the neuron would convey. If real neurons were adapting in such a way, this procedure would then predict how their filters would change with the stimulus distribution. We made the following assumptions: **(i)** the linear filter was biphasic, with a “fast” lobe of amplitude 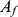, and a “slow” lobe of amplitude 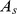, whose widths and the positions were fixed, so the filter was fully specified by the two amplitude parameters 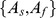 as schematized in Fig. 10A (also see Methods); **(ii)** we maximized the information rate 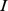 for a fixed average firing rate 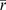; **(iii)** the effective noise of the neuron, or fraction of output entropy that is lost to noise, 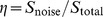, was fixed. We chose biphasic filters for three reasons: first, they can be parametrized easily, making their optimization tractable; second, the filters of retinal ganglion cells in different species and across stimuli have bi- or mono-phasic shapes; third, on such filters the balance index 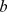 is well-defined and interpretable, a condition for comparing 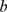 extracted from data to our model findings. We also note that 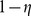 is a standard measure of coding efficiency. 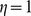 means that all the entropy of the spike train is noise entropy, i.e., that the response is completely uncorrelated with the stimulus; such a code has zero coding efficiency. On the other hand, 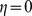 means that the total entropy of the spike train codes for information reliably, corresponding to a 100% efficient code. In our case, the total and noise entropies are estimated as in Ref [49].

Figure 10 shows the dependence of the information rate on the shape of the stimulus filter: left-skew distributions (S−−) slightly favor OFF cells (negative fast lobe, positive slow lobe, Fig. 10B), while right-skew distributions (S++) slightly favor ON cells (not shown). This conclusion was robust to noise in the neuron 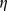 ranging from 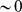 to 0.5, which is broadly the range for salamander ganglion cells [36]. For a specific choice of 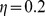 in Fig. 10C, Fig. 10D shows the dependence of information rate on the ratio of the fast to slow lobe amplitude, 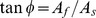. For each stimulus (S++ and S−−), there are two local maxima, one for an ON-like and one for an OFF-like cell, which differ in the transmitted information by less than 10%. Importantly, however, the maxima are not achieved at the same value of 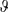 in both stimulus conditions – this means that while an adapting ON or OFF cell can maintain the same rate of information transmission when the stimulus changes, it will need to modify the filter shape by adjusting the ratio 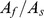.

Focusing on the case of an OFF cell, we found that an optimally adapting cell would increase the area under the fast (negative) lobe and would decrease the area under the slow (positive) lobe with increasing skewness. We used the balance ratio 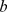, introduced previously, to quantify this change. Figure 10E shows significant changes in the filter shape with skew that range from 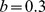 for S−− to 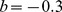 for S++ (the optimal filter for the Gaussian stimulus is balanced (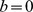), with equal and opposite areas under the two lobes). However, these substantial changes in the filter shape only lead to moderate changes in the amount of encoded information: in the case shown in Fig. 10, the information gain of the adaptive neuron relative to the case of no adaptation is always less than 10%. While the exact number varies with the chosen constraints—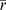, 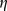, the locations and widths of the filter lobes—three qualitative observations remain true: **(i)** we have theoretically shown that biphasic filters outperform monophasic (single lobe) filters for all skewed and Gaussian stimulus ensembles examined; **(ii)** the neurons should adjust the biphasic linear filter away from the balanced configuration (optimal for Gaussian stimuli) by systematically adjusting the weight under the fast and slow lobes so that, e.g. in case of the OFF cell, negative skew favors larger weight in the slow lobe; but also that **(iii)** these adaptive changes would only lead to small relative information gains.

The optimal changes in the filter shape predicted here for a change in skewness are qualitatively consistent with changes in real filters observed in Fig. 4: for OFF cells, positive skewness leads to negative 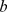, and negative skewness leads to positive 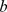, although the changes observed in the recordings are smaller than the optimally predicted ones. Despite this apparent match, we find that neither in the theoretical model nor in the data do changes in filter shape lead to very large changes in the amount of information a neuron can encode about the stimulus.

## Discussion

We explored adaptation to higher-order statistics in the light signal, by analyzing the responses of salamander retinal ganglion cells to temporally uncorrelated and spatially uniform stimuli with Gaussian, skewed and kurtotic luminance distributions. While the retina is highly adaptive to changes in first and second order statistics, we found much smaller changes in neural encoding in response to variations in stimulus skewness and kurtosis. A specific instance of this has been reported in relation to switches between a Gaussian and a binary stimulus of the same variance; there appeared to be no adaptation to kurtosis, but higher-order statistics could, interestingly, affect the dynamics of adaptation to contrast [50]. A related result was reported in the cat LGN for spatially structured stimuli, using 1D LN models inferred using reverse correlation [31]: no changes in cells' encoding properties were observed for spatial textures, but the range over which the skewness and kurtosis varied was much smaller than in our study.

To establish our result, we compared the shape of linear filters across the stimulus conditions directly (by measuring their overlap and the balance quantity 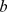), by information-theoretic measures (information per spike captured), and through the impact on the prediction performance; similarly, we assessed the changes in the nonlinearity by measuring their impact on the firing rate prediction performance. In all cases—with the possible exception of the highly kurtotic (K+) stimulus—we found that global models, i.e., models with an invariant pair of filters and an invariant nonlinearity, account for the neural behavior almost as well as the models fit to different stimuli separately. This is in stark opposition to contrast adaptation, where some encoding properties of the cells (e.g., nonlinearity gains) change in proportion to the change in contrast and are thus straightforward to detect.

Do the ganglion cells in the salamander retina adapt to skewness and kurtosis? Unlike for the case of contrast, this question is not easy to answer. We do observe statistically significant changes in the shape of filters between some, but not all of the conditions, as well as changes in the information captured by the model, but these changes are fractionally very small; some of them are close to the estimated resolution of our inference method. When one approximates a neuron responding to stimuli of varying statistics with a simple LN model, one would expect, on general grounds, that the parameters of the approximating LN model depend on the stimulus statistics. The observed changes thus do not necessitate the existence of a separate biological adaptation process in the real neuron, and could simply imply that the neuron's best LN approximation is slightly different when it is driven by signals with different statistics. Furthermore, adaptation is usually taken to mean not any change in encoding, but a *functional* change in particular, one which improves the neuron's signaling quality. When viewed through this measure, the small changes we observe improve information transmission only marginally over a non-adapting model. In sum, while our measurements cannot rule out adaptation to the list of higher-order statistics that we tested, they strongly suggest that any adaptive changes that can be accounted for by 2D LN models must be small and are unlikely to be functionally significant for encoding, with the possible exception of K+ stimulus.

Should the ganglion cells in the salamander retina adapt to higher-order statistics? Two analyses that we performed suggest that for temporally uncorrelated stimuli adaptation to HOS is unlikely to yield large benefits for information encoding. First, any model of neural processing which starts by linearly summing the light signal in the recent past with an invariant linear filter will tend to wash out any signatures of HOS, such that the output of the linear filter will converge to a Gaussian distribution, by direct application of the central limit theorem. This convergence will be much faster if the signal is spatio-temporal as in the case of Bonin et al. [31], since in that case the neurons perform spatio-temporal summation in their receptive field over a much larger number of samples from the luminance distribution. In short, after the linear stage, the only two statistics that are retained from the original signal in the distribution of filter outputs are the mean luminance and the contrast. Second, even if the linear filters are not invariant but can change with the stimulus condition, our computational model in Fig. 10 shows that substantial changes in optimal filter shape with skewness only yield order 10% improvements in encoded information or smaller, much less than what contrast adaptation can deliver (Fig. 8B). While the exact numbers are specific to model details, the general argument that makes contrast adaptation qualitatively different from the putative HOS adaptation remains true (but see the discussion about analysis limitations below).

If the central limit theorem erases signatures of higher order statistics in the filter outputs, could the nervous system beyond the retina ever respond to such statistics when manipulated in synthetic stimuli (e.g., as in the psychophysically demonstrated sensitivity to changes in luminance histograms beyond contrast in humans)? First, there might be cells that respond selectively to HOS which are rare and haven't been observed in our experiment. Another explanation is that in salamanders, and unlike in humans, there simply is no sensitivity to higher order moments of the luminance distribution. The third option is that the removal of HOS statistics by linear filtering is not complete, only approximate, and the remaining deviations get encoded into the spike trains. Lastly, there exists another interesting explanation. Central limit theorem guarantees that the distribution of filter outputs will converge to a Gaussian. However, linear filtering (even of identical independently distributed luminance levels) induces temporal correlations in the filter output, and consequently could induce temporal correlations in the spike train. Those correlations could differ between two stimulus distributions matched in mean and variance, and differing in HOS. In contrast, for natural stimuli with long temporal correlations, the linear filtering in the receptive field might not (fully) remove the signatures of HOS, as we explain below, thus allowing downstream processing to detect and respond to HOS changes.

Even if an adaptive code were to confer (a small) coding benefit, it would also incur additional costs due to its ambiguity. The same response from an adapting neuron can, for example, signal two different light intensities, depending on the stimulus history. Downstream neurons must therefore rely either on keeping track of the adaptive state of the encoding neuron, or on using diversity in the neural population in a proper way to estimate the stimulus. Moreover, nontrivial processing is required also on the encoding side: the adaptive neuron needs to infer from the stimulus itself whether some underlying property of the stimulus, such as the mean luminance or contrast, has changed and thus an adaptive response needs to be triggered. Such detection might not be easy, and in particular, might require a substantial number of independent stimulus examples (and thus time). In short, adaptive codes most likely incur computational costs that invariant codes do not. One explanation for the lack of dynamic adaptation to higher-order statistics is therefore that it does not yield much gain in information, while potentially increasing the coding cost and complexity. Instead of implementing a costly adaptation mechanism able to dynamically change the filter shape on an individual cell basis in response to stimulus skew, the neural population could, for example, be structurally adapted to the overall luminance distribution of natural scenes, by (e.g.) properly partitioning the population between ON- and OFF-like cells [51].

Taken together, while some of the (small) changes in the encoding properties of retinal ganglion cells that we report may be statistically significant, the issue of whether these changes constitute “adaptation” is likely to remain a matter of interpretation. The changes are consistent with adaptation, and are also qualitatively consistent with the theoretically expected changes in optimal filter shape for different values of skewness. On the other hand, their functional effect on information encoding is small compared to the changes during, and benefits of, the adaptation to contrast. This is expected based on a simple theoretical argument, and confirmed in a toy model of an adapting LN neuron. Sparse, highly kurtotic stimuli (like K+) merit further attention, and point to the possibility that an alternative experimental design could be more successful in pursuing adaptation to HOS. To be concrete, we discuss below three limitations of our current analysis and conclude by making suggestions for follow-up experiments.

First, we have used LN models fitted to data to probe for adaptation, by looking for stimulus-statistics-induced changes in the model parameters. While this is a standard procedure in sensory neuroscience, we note that one cannot *exclude* adaptation if LN model parameters don't change, or if they change very slightly. If information were encoded in, for example, spike-train temporal correlations, and the neuron modulated that correlation structure with the stimulus condition, our analysis would have missed such changes. One could attempt to capture the effects of spike interactions by, e.g., generalized linear models [43] or Keat-type models [35], which would also enable us to predict the PSTH of single neurons even better. Alternatively, adaptation could be taking place at the level of the whole neural population, an idea that we explored recently in a theoretical setting [52]; our current analysis would also be unable to capture such effects. Nevertheless, on the level of single-spike sensitivity to the stimulus, our 2D LN models tend to capture the majority of information per spike, and it is unlikely that any major adaptive effects would go unnoticed.

Second, we were restricted to the low range of contrasts by the limitations of our display device. If HOS adaptation mechanisms were conditioned on the light signals having a high contrast, our current experimental design would preclude us from detecting such adaptation. More fundamentally, it is not clear what are the actual statistics that the neurons are adapting to [31], [53]. While we commonly think in terms of contrast, skew, and kurtosis as the relevant statistical properties of stimuli, it is not obvious that the brain relies on these same measures in dealing with natural scenes. In particular, they may be poor choices in natural settings, as their values are sensitive to outliers and because they might vary in a dependent way in nature (c.f. [54]). It therefore remains an open question which estimators the retina (and other neural systems) are using for contrast-, skew-, and kurtosis-like statistics, and which of these measures drive adaptive processes.

Third, our reasoning about the limited benefits of HOS adaptation in LN-type neurons rests on the application of the central limit theorem. We used full field stimuli (which are spatially maximally correlated) to address an absence of the observed adaptation in Ref [31]. There, random checkerboard stimuli were used, raising the possibility that adaptation was not observed because the convergence to a gaussian distribution after linear summation in the receptive field is much faster. Despite strong spatial correlations in our full-field flicker, we don't observe a large adaptive effect, and thus an interesting extension to our analysis would consist of using spatially homogenous and temporally correlated stimuli, or of using heavy-tailed (or naturalistic) luminance histograms – if they can be reproduced in the lab using display hardware with a larger dynamic range [55]. With such a display it would also be interesting to explore in detail the responses to sparse kurtotic (K+) type stimuli, the only stimulus ensemble for which the performance of global models was noticeably lower. Both of these extensions would affect the central limit theorem argument above: in the case of temporally correlated stimulus, the samples would no longer be independently drawn and thus might not (quickly) converge to a Gaussian; in case of heavy tails, the linear filter similarly might not be able to average over a sufficient number of luminance samples to “erase” the signatures of higher-order statistics. A concrete hypothesis to test in a future experiment would therefore be to ask whether the shape of the nonlinearity in a ganglion cell can adapt to the distribution of linear filter outputs, even when those are no longer Gaussian. This is a test that should especially be relevant for responses of ganglion cells to natural movie clips. As both proposed stimulus distributions would bring the stimuli closer to the naturalistic ones, they could provide us with a more complete window into the nature and limits of retinal adaptation to natural scenes.
